# Supplementary material for: Reproductive healthcare in prison: A qualitative study of women’s experiences and perspectives in Ontario, Canada
Source: PLoS One. 2021 May 18;16(5):e0251853. doi: 10.1371/journal.pone.0251853 (PMC8130921; doi:10.1371/journal.pone.0251853)
Supplement: Attachment — Submitted filename: Response to Reviewers.docx [file pone.0251853.s002.docx]

**Response to reviewers**

**EDITOR COMMENTS:**

1. Please submit a completed COREQ checklist with the revised manuscript. Ensuring compliance with the Equator Network guidelines helps with methodological rigor and reporting.

**Response:** A COREQ checklist has been submitted with the revision.

2. Consider moving beyond a summative approach to the qualitative data to a more theoretically driven analysis. As the reviewers note, a reproductive justice framework might be a useful framing for the paper, although there are other frameworks that would help to guide interpretation of the data. I also agree with reviewer #2 who suggested that presenting the quotes in tables makes it difficult to read and connect with the text.

**Response:** We have revised and extended our analysis using a deductive content analysis approach and a reproductive justice framework (see the new ‘analysis’ section within Methods). We have added the quotes, and have removed the tables, into the text to facilitate connection. We thank the reviewers for these suggestions which we feel have strengthened the paper.

3. Given the extended time period and multiple settings, the methods section should include some justification for analyzing these data together. Were the two sets of qualitative data analyzed separately, and similar themes were encountered? Was saturation achieved? Being explicit in the analysis about which themes were identified by the participants residing in the community and which by currently incarcerated participants may help with this as well.

**Response:** Thank you for this comment. We have removed the data from the community-based focus groups to improve clarity. The paper now reports data from the in-prison focus groups only.

4. The manuscript is somewhat disjointed, which I attribute to a lack of theoretical underpinning. Framing from the introduction in a theoretically motivated way will allow for carry through into the analysis and reporting of the results and discussion of the findings.

**Response:** We have modified all sections of the paper to be more cohesive around a reproductive justice framework, as described in the Analysis section of the Methods.

**JOURNAL REQUIREMENTS**

1. Please ensure that your manuscript meets PLOS ONE's style requirements, including those for file naming. The PLOS ONE style templates can be found at

https://journals.plos.org/plosone/s/file?id=wjVg/PLOSOne_formatting_sample_main_body.pdf and

https://journals.plos.org/plosone/s/file?id=ba62/PLOSOne_formatting_sample_title_authors_affiliations.pdf

**Response:** Thank you. We have adjusted the manuscript to meet the style requirements.

2. Please include a copy of the interview guide used in the study, in both the original language and English, as Supporting Information, or include a citation if it has been published previously.

**Response:** We have added the focus group guide as supporting information.

3. Please provide additional information regarding the considerations made for the prisoners included in this study. For instance, please discuss whether participants were able to opt out of the study and whether individuals who did not participate receive the same treatment offered to participants.

**Response:** We have added the following text “*Participants were informed that they were able to opt out of the study at any time, and that participation in the study would not impact their treatment in the facility.”* (line 148-149).

4. Thank you for stating the following in the Competing Interests section:

"The authors have declared that no competing interests exist."

We note that one or more of the authors are employed by a commercial company: Independent Researcher.

4.1. Please provide an amended Funding Statement declaring this commercial affiliation, as well as a statement regarding the Role of Funders in your study. If the funding organization did not play a role in the study design, data collection and analysis, decision to publish, or preparation of the manuscript and only provided financial support in the form of authors' salaries and/or research materials, please review your statements relating to the author contributions, and ensure you have specifically and accurately indicated the role(s) that these authors had in your study. You can update author roles in the Author Contributions section of the online submission form.

Please also include the following statement within your amended Funding Statement.

“The funder provided support in the form of salaries for authors [insert relevant initials], but did not have any additional role in the study design, data collection and analysis, decision to publish, or preparation of the manuscript. The specific roles of these authors are articulated in the ‘author contributions’ section.”

If your commercial affiliation did play a role in your study, please state and explain this role within your updated Funding Statement.

**Response:** The author listed as ‘Independent Researcher’ is currently employed by a public health unit but did not participate in this project in this role, so we did not list this organization as their affiliation. This author was not paid to do any of the work involved in this project by any employer (past or present) nor did they receive any of the project’s funding in return for their services. We have clarified this in the funding statement (see cover letter). Please let us know if it would preferable to list the author’s location (e.g., Dundas, Ontario), as opposed to an affiliation (i.e. ‘Independent Research’) in this circumstance.

4.2. Please also provide an updated Competing Interests Statement declaring this commercial affiliation along with any other relevant declarations relating to employment, consultancy, patents, products in development, or marketed products, etc.

Within your Competing Interests Statement, please confirm that this commercial affiliation does not alter your adherence to all PLOS ONE policies on sharing data and materials by including the following statement: "This does not alter our adherence to PLOS ONE policies on sharing data and materials.” (as detailed online in our guide for authors http://journals.plos.org/plosone/s/competing-interests) . If this adherence statement is not accurate and there are restrictions on sharing of data and/or materials, please state these. Please note that we cannot proceed with consideration of your article until this information has been declared.

Please include both an updated Funding Statement and Competing Interests Statement in your cover letter. We will change the online submission form on your behalf.

**Response:** Thank you. We have updated the Funding Statement and Competing Interests Statement in our cover letter.

Please know it is PLOS ONE policy for corresponding authors to declare, on behalf of all authors, all potential competing interests for the purposes of transparency. PLOS defines a competing interest as anything that interferes with, or could reasonably be perceived as interfering with, the full and objective presentation, peer review, editorial decision-making, or publication of research or non-research articles submitted to one of the journals. Competing interests can be financial or non-financial, professional, or personal. Competing interests can arise in relationship to an organization or another person. Please follow this link to our website for more details on competing interests: http://journals.plos.org/plosone/s/competing-interests

5. Please include captions for your Supporting Information files at the end of your manuscript, and update any in-text citations to match accordingly. Please see our Supporting Information guidelines for more information: http://journals.plos.org/plosone/s/supporting-information.

**Response:** This has been added.

**REVIEWER COMMENTS**

**Reviewer #1:** Thank you for the opportunity to review this manuscript on an important topic. Your background on the provincial prisons is helpful and contributes to our understanding of the context. I have two major and two minor comments.

A minor comment on page 6 is about including women aged 15 to 49 years. Can you clarify if juveniles are incarcerated in the provincial prisons—it would seem that women 15-17 years old would be in juvenile facilities. Perhaps include this in the description of provincial prisons.

**Response:** We have corrected our original error. While earlier versions of our protocol included 15-49 years old, our final inclusion criteria used in recruitment materials, and the minimum age range for women admitted to the facility, was 18 years old (line 135-136).

Page 7, line 147 needs a citation.

**Response:** We have clarified the description of our approach and have added a reference (Elo S, Kyngas H. The qualitative content analysis process. J Adv Nurs 2008; 62(1): 107-15, specified in ‘Overall Approach’ and ‘Analysis’ (line 69, 159, respectively).

One major concern is that lack of any type of social or reproductive justice framework in which to analyze and apply findings. If the authors might consider retrospectively applying one, I think it would address both of the below concerns

**Response:** Thank you for this suggestion. We have applied a reproductive justice framework, as described in the Analysis section of the Methods.

Of the five themes that were identified one seems out of place. From the title, “Contraception and reproductive healthcare in prison”, the theme "the importance of contraception and barriers to access for women in prison” and its related quotes seem out of place. The quotes are not about health care, they are about how women in the target population feel about contraception; this would seem to be a separate topic. I found the theme and quotes distracting and out of sync with the rest of the themes and suggest deleting.

**Response:** We agree with the reviewer, and have deleted the content and quotes dealing with how women in the target population feel about and challenges with specific types of contraception. This is described in ‘Results’ (lines 194-196).

On page 23, I believe that a final statement is weak, “Our findings can inform interventions to improve the quality and accessibility of healthcare in prison and post-release and support the development of programs that make essential services such as contraception a routine part of the care of women in prison.” The quotes in the manuscript are so disturbing, surely we want to do more with them than “inform interventions”. I do not mean to impose on own agenda on the authors, but suggest that the reason we do this type of research is to use the voices of women in this population to make changes. A stronger final statement would be appropriate.

**Response:** We agree and have changed the sentences to: *Our findings can inform interventions to improve the quality of reproductive healthcare in prison in ways that “disrupt the dehumanizing status quo of reproductive politics” and promote reproductive justice, which includes equity, freedom and dignity (page 11) [40].* (lines 516-518).

**Reviewer #2:** Abstract: Too general e.g., 'structural and cultural barriers.' Share specific findings from paper.

**Response:** We have modified the abstract to include more specific results: “*The data reflected three components of a reproductive justice framework: 1) women have limited access to healthcare in prison, 2) reproductive safety and dignity influence attitudes toward pregnancy and contraception, and 3) women in prison want better reproductive healthcare. Discrimination and stigma were commonly invoked throughout women’s experiences in seeking reproductive healthcare.”* (lines 32-36).

Introduction: Need to better define the frame of reference for this study. It starts with women worldwide, but most of the cites about access to services appear to refer to studies in North America. Later, the rationale is presented that it is important to know about women in Canada because there is a national health system. In this case, the background presented should refer to incarcerated women in Canada or incarcerated women in countries with national health systems.

**Response:** We have modified our introduction (i.e. ‘Background’) in keeping with reviewer suggestions to incorporate a reproductive justice framework. In doing so, we have referenced evidence from multiple jurisdictions (e.g., Canada, Australia, and the United States), since we believe this evidence is at least partly generalizable to our target population, specifically when considering a reproductive justice framework.

Methods: Sample is weak - women recruited from two different types of venues two years apart. Qualitative analysis methods inadequately described.

**Response:** We have removed the community-based sample, and now only include focus groups conducted with women in prison in 2018. We have improved the description of our qualitative methods, specifying a combined deductive and inductive content analysis approach focused on a factist philosophy and the manifest data content (lines 68-74, and 158-173).

Results: results are organized by topic area, but not by theme as the authors state. For example, gatekeeping by prison personnel would be a theme. "Perspectives on pregnancy" is a topic. There is very little thematic analysis in this paper.

**Response:** We have clarified that we report categories of data which were synthesized in a concept map (Figure 1), in keeping with content analysis methodology. We have reorganized and modified the results section accordingly.

Results shift back and forth between what the women experience in prison and what the women experience more generally. This weakens the uniqueness of the paper, which is what happens around reproductive health while IN prison.

**Response:** We agree, and have decided to focus on what women experience in prison, and the content which pertains to a reproductive justice framework. We have removed the data from the focus groups conducted in the community, and have modified the results accordingly.

Quotes should be integrated in text near the findings you describe, not grouped together in boxes. “Illustrative quotes” is not a term typically used in qualitative analysis. Some of the quotes don’t link up with any of the findings you discuss.

**Response:** We have integrated the quotes into the text to follow the description of the relevant findings.

The paper is lacking in analysis and insight. The conclusion, that incarcerated women should be given access to reproductive health care, is true but not unique and not directly tied to most of the findings that the authors present.

**Response:** We have modified our conclusion to emphasize both that women experiencing imprisonment should have better access to healthcare, but also that discrimination and stigma felt by women must be addressed in order to promote reproductive justice for women in prison. We believe this conclusion is more closely tied to our findings, and that it makes the main message of our paper more unique.

**Reviewer #3:** The article is an important contribution to the literature on incarcerated women's health and access to reproductive healthcare during imprisonment. There are two areas where this article can be strengthened and improved. One, the authors neglect to cite literature on family planning for incarcerated and formerly incarcerated women. Doing so would further substantiate some of the findings in this study.

**Response:** We have cited the following papers on family planning for women who experience imprisonment:

Clarke JG, Hebert MR, Rosengard C, Rose JS, DaSilva KM, Stein MD. Reproductive health care and family planning needs among incarcerated women. *Am J Public Health* 2006; **96**(5): 834-9.

Peart MS, Knittel AK. Contraception need and available services among incarcerated women in the United States: a systematic review. *Contracept Reprod Med* 2020; **5**: 2.

Sufrin CB, Creinin MD, Chang JC. Contraception services for incarcerated women: a national survey of correctional health providers. *Contraception* 2009; **80**(6): 561-5.

Schonberg D, Bennett AH, Sufrin C, Karasz A, Gold M. What Women Want: A Qualitative Study of Contraception in Jail. *Am J Public Health* 2015; **105**(11): 2269-74.

Schonberg D, Bennett AH, Gold M. The contraceptive needs and pregnancy desires of women after incarceration: A qualitative study. *Contraception* 2020; **101**(3): 194-8.

Second, the authors should also address potential ethical issues that are relevant when conducting studies in prison and how they addressed those ethical issues in their work. For example, receiving IRB approval is the bare minimum standard. What ethical issues did they consider prior to launching their work and how did they resolve them to proceed with this work.

**Response:** We have included a more detailed description of our study procedures, which outlines the issues we faced in proceeding with this work, under ‘Study Development’ (lines 94-121).
