# Supplementary material for: Reproductive healthcare in prison: A qualitative study of women’s experiences and perspectives in Ontario, Canada
Source: PLoS One. 2021 May 18;16(5):e0251853. doi: 10.1371/journal.pone.0251853 (PMC8130921; doi:10.1371/journal.pone.0251853)
Supplement: Attachment — Submitted filename: Response to Reviewers_April 27.docx [file pone.0251853.s003.docx]

**Reviewer #1: Really lovely revision. I have two minor comments. The first is to ask for a bit of clarification about the topic of accessing contraception while incarcerated. Readers may wonder why is this necessary--you have provided one strong rationale about continuity (starting while incarcerated will make it easier to continue when out), but some additional text on this would be helpful. Also, why women would need condoms while locked up needs some explanation.**

Response: Thank you for this feedback. We have added the following content to the Background:

“The World Health Organization and the United Nations recommend the provision of reproductive healthcare, specifically including contraception and pregnancy-related care, for women in prison [14]. Access to contraception in prison can support women in preventing unintended pregnancy after release, which is particularly important since women may be at increased risk of unintended pregnancy during that period and they often face barriers to contraception access in the community, including urgent competing priorities while transitioning back to the community [15]. Contraception access in prison is also important for females who are sexually active with males in prison.” (Lines 54-61).

References 14 and 15 were added to support these points.

**Second, I find the role of correctional officers as gatekeepers to health services horrifying and I would suggest that the topic of reproductive health care be considered under the umbrella of overall health care. A final plea/recommendation might be along the lines that women are whole persons deserving of health care, including reproductive health services. While the focus is reproductive health, we want all of the health services for women to be improved.

Once again, nice job!**

Response: Thank you, we agree with highlighting the importance of improving health services for women overall. We have added the following italicized content to the discussion:

“Our results also raise the question, as posed by other reproductive justice scholars [40], as to whether reproductive justice can in fact be achieved in prison since these data suggest that women’s desires to have better quality and access to reproductive healthcare, *and healthcare overall,* has largely been negatively impacted by the conditions of their arrest and incarceration. *Ultimately, although our study was focused on reproductive healthcare, our findings highlight ways in which systemic changes are necessary to promote the overall health of women in prison*.” (Lines 518-524).

**Reviewer #2: I appreciate the effort the authors put into revising the paper and it is greatly improved as a result. I have a few remaining, fairly minor comments:

Background: Situate the literature cited. Is it from North America? Western Countries? Worldwide?**

Response: We have specified where the cited literature is situated throughout the Background. Most of the literature is from North America, although some references are based on research in other areas. We also removed one reference (Covington, S.S., *Women and the criminal justice system.* Womens Health Issues, 2007. **17**(4): p. 180-2.) since it was an editorial without original data.

**Approach lines 68-71: Unclear what the authors means when they say they did not 'assume a pre-specified framework' in order to 'build off previous work in this area.' Are they referring to the authors’ previous work or the literature? It seems like building off previous work would contribute to specifying a theoretical framework. Clarify.**

Response: We had originally meant building off our previous work in this area, but on reviewing this line again we agree with reviewer that it was unclear, so we removed “to build off previous work in this area” from this section (Lines 77-79).

**Study development section: While it’s helpful for the reviewers who saw the first draft of the paper, I don’t it's necessary to include the information about the community sample, since you are not using the data. The summary of quantitative results is a good addition.**

Response: We agree with this suggestion, and have removed the information about the community sample (Lines 120-123).

**Line 134: Where were flyers posted (common areas? restrooms?) and announcements made?**

Response: We have added that flyers and announcements were made in common areas in the prison (Line 136-137).

**Lines 139-146: Too much detail about the facilitator. I would restrict the description of the facilitator to say they had training in qualitative methods and feminist epistemologies.**

Response: We have shortened the description as suggested:

“The focus groups were facilitated by one female team member (JJ), who had graduate-level training in qualitative methods and feminist epistemologies.” (Lines 142-143).

**Line 150: Were any 'ground rules' discussed for focus groups? (eg, confidentiality among participants)**

Response: We have added the following details (italicized below) about the ground rules discussed during the focus groups:

“In the letter of information participants were informed that they were able to opt out of the study at any time, and that participation in the study would not impact their treatment in the facility. *They were asked to be respectful of each other’s experiences given the sensitive and personal nature of the conversations and to keep what was shared in the group confidential and were also reminded that confidentiality could not be guaranteed following the group. The facilitator reviewed the letter of information and consent form verbally* and also made participants aware of her affiliation, research background, context of the current research as situated in the previous research completed on the topic by the researchers, and the objectives of the research.” (Lines 146-154).

**Lines 166-168: The reference to the previous set of reviews is likely to be confusing to a new reader.**

Response: We agree with this feedback and have simplified this section to the following:

“After initial review of our results, we considered using a reproductive justice framework to enhance our analysis and interpretation” (Lines 170-171).

**Lines 168-172: Most of this belongs in the results section. Here, I would simply say that you used a Reproductive Justice Framework with a citation.**

Response: Thank you for this feedback. We agree and have moved this section to Results (Lines 188-192). We changed this to say we used a reproductive justice framework, with a citation as suggested (Lines 170-171).

**Line 180: How many women were in each focus group?**

Response: We have clarified the number of women in each group, by modifying this sentence to the following:

“We conducted three focus groups with seven women in each group, for a total of 21 participants in a provincial prison.” (Line 179).

**Lines 188-193: The discussion of subcategories is confusing, in terms of which belong to which larger categories. It may be clearer to simply refer to the figure.**

Response: We have made the suggested changes:

“Within these categories, the data suggested four sub-categories (see Figure 1).” (Line 187-188).

**Line 200: Mention why this subcategory was not included in the final analysis**

Response: We have added the following to our caption for Figure 1, and this is also explained in the Results section (Lines 196-198).

“Sub-category ‘challenges with contraception’ was not included in our final analysis in order to streamline results, as the relevant concepts within this sub-category were captured within the other categories.” (Lines 245-247).

**The reorganization of quotes to support areas of analysis has strengthened the paper tremendously. They now have a lot more impact

Line 415: quote a little confusing. Who are 'they'? Establish context.**

Response: We clarified that ‘they’ referred to correctional officers (Line 420).

**Discussion: I would love to see a paragraph highlighting the excellent and concrete ideas participants shared for improving reproductive health care in the prison setting. I highlighted a few as I read:**

**contraception should be discussed during routine release planning
contraception should be discussed and offered during the routine nursing assessment on prison admission
staff should ask women about their current needs for and interest in contraception**

Response: Thank you for this suggestion. We added the following to the Discussion:

“Participants had several suggestions to improve reproductive healthcare in prison, including making information regarding contraceptive options more readily available prior to seeing a doctor, asking women about contraceptive preferences on admission to the prison, and improving continuity with services in the community to access contraception.” (Lines 529-533).

**I would also like to see the issue of miscarriage highlighted a bit in the discussion, as it seemed important to women's experiences and simple interventions like providing sufficient access to menstrual pads could reduce misery and stigma**

Response: We agree that pregnancy loss was an important issue to highlight. We have added the following text to the Discussion:

“In particular, with respect to pregnancy, participants shared how lack of access to adequate healthcare and health resources contributed to trauma surrounding pregnancy loss or the potential for pregnancy loss while being detained.” (Lines 453-455).

**Reviewer #3: It appears that the authors have adequately responded to the reviewers concerns. I think this article will make a meaningful contribution to the literature.**

Response: Thank you!
